# The Effects of Chess Instruction on Pupils' Cognitive and Academic Skills: State of the Art and Theoretical Challenges

**DOI:** 10.3389/fpsyg.2017.00238

**Published:** 2017-02-23

**Authors:** Giovanni Sala, John P. Foley, Fernand Gobet

**Affiliations:** ^1^Department of Psychological Sciences, University of LiverpoolLiverpool, UK; ^2^Chess in School & CommunitiesLondon, UK

**Keywords:** chess, cognition, academic achievement, meta-analysis, placebo effect

## Introduction

Chess instruction has been claimed to enhance primary and middle school students' mathematical abilities. The “Chess Effect” hypothesis has received some scientific support but it is yet to be convincingly demonstrated. This note briefly reviews the prevailing research, identifies some common pitfalls, and recommends directions for future research.

Mathematics proficiency is seen as a necessary prerequisite for gaining jobs in the Science, Technology, Engineering and Mathematics (STEM) disciplines, which underpin our technological future. While the level of the required mathematical skills is increasing, the global educational surveys PISA and TIMSS have documented striking differences in proficiency levels between countries, which have created concern in several countries on their relative performance in mathematics. For example, from the USA perspective, researchers have conducted comparative analyses of performance trends (Hanushek et al., 2012) and also of mathematics pedagogy (Richland et al., 2012). There is a general feeling that novel methods of teaching have to be developed to make mathematics instruction more effective.

Chess instruction in school has been proposed as an intervention to address this objective. The conventional wisdom that chess instruction may enhance pupils' academic performance has stimulated numerous research projects worldwide over the last two decades. Most of the studies have focused on the putative benefits of chess instruction on achievement in mathematics.

## A meta-analysis of the available evidence

A recent meta-analysis has evaluated the effectiveness of chess instruction (Sala and Gobet, 2016). The meta-analysis, including 24 studies and 40 effect sizes, shows that chess does seem to enhance primary and middle school students' achievement in mathematics (*d̄* = 0.38) and overall cognitive ability (*d̄* = 0.34). The effects on achievement in literacy appear to be modest (*d̄* = 0.25). Moreover, the size of the effects is positively related to the amount of training, suggesting that 25–30 h, equivalent to a lesson per week during the school year, is probably the minimum threshold to obtain meaningful benefits. However, in spite of the promising results, this meta-analysis also points out that almost none of the reviewed studies compared chess-treated groups with active control groups to rule out possible placebo effects. At present, this is the most serious methodological issue in the field.

## The IoE study

A new study by the Institute of Education, London (the “IoE study”) has challenged the Chess Effect hypothesis (Jerrim et al., 2016). The study compared a large group of Year 5 pupils (age 9/10) (*N* = 1,965) engaging in 1 year of chess instruction (25–30 h) with a passive control group of peers (*N* = 1,900). The school classes were randomly assigned to the two groups and pre-tested via Key Stage 1 public examinations for literacy, science, and mathematics. One year after the end of the treatment, the participants were post-tested via Key Stage 2 public examinations in the same disciplines. The two groups did not differ in any of the outcome measures. This result attracted some attention from the UK press (e.g., Pells, 2016) because it contradicts not only the previous research, but also the common view of many head teachers and educationists about the presumed benefits of chess. For these reasons, this study is worth some further discussion.

The IoE study possesses some strengths: a large sample, administrative data for both the pre-test and the post-test, and group allocation by randomization. However, there are two major weaknesses in the experimental design that lead us to doubt the reliability of the null results, especially in relation to mathematics. First, as previously mentioned, the post-test was administered 1 year after the end of the instruction. Thus, the IoE study sought to derive the “long-term” impact of chess instruction. However, the lack of an immediate post-intervention measure allows no direct comparison with previous studies, as the literature has focused on the short-term impact. Previous research indicates that 25–30 h of instruction and play is the minimum required to gain a significant short-term impact (Sala and Gobet, 2016). Hence, it is improbable that the same amount can induce a long-term impact. Second, the results may be vitiated by a ceiling effect. The IoE study reported an overall mean of 70% and a standard deviation 20% in Key Stage 2 mathematics. Moreover, the distribution was highly negatively skewed (Jerrim et al., 2016, Figure 2, p. 27), with approximately half of the sample performing above 75%. These sample scores are consistent with those at the national level published by the Department for Education (for details, see Statistics: key stage 2, 2017^1^. Given that the exam system generates an artificially constrained distribution of test results, measuring the effect of any intervention is problematic.

## Unresolved issues of the research on the benefits of chess instruction

Even if the IoE study does not provide any clear evidence against the alleged benefits of chess instruction, the evidence produced so far is insufficient to establish those benefits either. Some essential design-related and theoretical questions are yet to be resolved.

### The problem of placebo effects

Apart from this single IoE study, previous research on the effects of chess instruction plausibly indicates a positive impact on mathematics performance. However, we cannot rule out placebo effects because almost none of the experiments in the field of chess and education were designed with active control groups. (The exception is Sala et al., 2016, where the chess group was compared to both a passive control group and a group playing go.) All the qualitative analyses, including the IoE Study, show that most pupils are enthusiastic about chess. This enthusiasm may make the pupils more motivated about school—which in turn boosts academic performance. Many other activities may be as motivating as chess and, hence, obtain the same positive results.

The necessity of active control groups to control for placebo effects goes beyond the particular case of chess and encompasses training interventions in general (Moreau et al., 2016). Crucially, it has been recently observed that the type of control group (active or passive) is often a significant moderator in meta-analytical models. For example, comparisons between treatment and active control groups systematically provide smaller effect sizes than comparisons between treatment and passive control groups in domains such as working memory training and music instruction (Melby-Lervåg et al., 2016; Sala and Gobet, 2017a).

### The lack of a cognitive link

The generalization of chess skill to the domain of mathematics would be an example of *far transfer*. Far transfer occurs when a set of skills generalizes across domains only loosely related to each other (Thorndike and Woodworth, 1901; Anderson, 1990; Barnett and Ceci, 2002). Substantial research on transfer has strongly suggested that far transfer occurs, but rarely and with minimal effects (Donovan et al., 1999; Gobet, 2016). Examples of failed far transfer include music instruction to improve children's (aged 3–14) cognitive ability or academic achievement (Sala and Gobet, 2017a) and working memory training to enhance overall cognitive ability (Melby-Lervåg et al., 2016; Sala and Gobet, 2017b).

So, why should chess instruction improve academic performance? The proposed explanations refer to the fact that chess is a cognitively demanding activity. Chess requires domain-general cognitive abilities that may be trained by the practice of the game. Then, those cognitive abilities may transfer to other domains. For example, Bart (2014) suggests that chess involves, and possibly boosts, cognitive abilities such as working memory, fluid intelligence, and concentration capacity (see also Burgoyne et al., 2016; Sala et al., in press). These abilities are predictors of achievement in mathematics (e.g., Deary et al., 2007; Peng et al., 2016), which would explain why chess increases pupils' mathematical ability. A similar argument is deployed in the IoE Study (Jerrim et al., 2016; p. 6 *et seq*.). Chess may be beneficial for mathematical ability and, more widely, academic achievement by enhancing concentration and problem-solving skills.

These explanations, albeit lacking detail, are plausible and provide the basis for the hypothesis that chess instruction strengthens cognitive abilities that are positively correlated to achievements in mathematics. Unfortunately, only a few studies have investigated the effects of chess on both cognitive abilities and academic outcomes. The results so far have been mixed (Scholz et al., 2008; Sala et al., 2016). In brief, the causal mechanisms remain substantially untested.

## Recommendations for future research

Combining the research results so far, we may conclude that exposure to chess instruction is associated with positive results in mathematics performance in the general population of primary and middle school students in the short term but not in the long term. Consequently, the validation of chess as an educational tool must undergo further research. A rigorous experimental design is needed to shed some light on (a) the potential placebo effects of chess instruction, (b) the cognitive mechanisms underlying the transfer from chess to mathematics skills, and (c) the appropriate type and duration of the teaching for this transfer to occur.

An active control group is necessary to understand whether the observed impact on pupils' achievement in mathematics is chess-specific or due to placebo effects (Gobet and Campitelli, 2006). Chess could be matched against another enrichment activity such as music or drama lessons. However, such a design would not rule out the possibility that both the treatments are equally effective for treatment-specific reasons and not just for placebo effects (e.g., because chess instruction enhances fluid intelligence and music training enhances spatial skills).

Another option is to compare the effects of chess with and without instruction. While exposure to unstructured chess activities (e.g., free play with peers) is not supposed to provide any particular benefit apart from placebo effects, a set of chess activities specifically designed to train cognitive/academic skills may be more effective. This design is the equivalent of the one often used in the field of working memory training (e.g., Jaeggi et al., 2011), where the effects of treatment are compared to the ones exerted by a non-adaptive version of the training program. The exposure of both the groups to the same stimuli (e.g., chess boards, pieces, playing games) guarantees the isolation of the placebo effects. Moreover, using two different versions of the same activity allows the same person(s) to deliver the treatment to both the groups, instead of a chess instructor for the chess group and another expert for the active control group. The advantage of this approach is that it allows us to control for possible Pygmalion effects.

With regard to the cognitive benefits of chess instruction, the empirical evidence is quite sparse. Future studies should investigate the effects of chess instruction on a wide set of cognitive skills related to mathematics, such as fluid intelligence, planning, working memory, and spatial ability. Multivariate measures of mathematical ability would help to understand the particular mathematical skills enhanced by chess instruction (e.g., logical analysis, problem-solving, arithmetic, geometry). A well-defined and testable causal model linking chess, cognitive and academic skills is needed. Such a model is essential to tailor effective methods for chess instruction.

The didactic methods in the teaching experiment should fulfill the requirements of common elements across domains for transfer to occur. Hence, they should incorporate those features that chess shares with mathematics such as the geometry of tactical patterns, the exchange value of pieces and problem-solving situations (Root, 2008; Sala et al., 2015; Trinchero and Sala, 2016). Examining various measures of chess skills (e.g., piece positioning, tactics, strategy) is required to link specific chess activities to the particular cognitive/academic skills. A systematic measuring scheme is required relating the quantum of chess instruction (e.g. 15, 30, 45 h, etc.) and the duration of the effect (0, 6, 12 months, etc.). Such a design would make a major contribution to our comprehension of the Chess Effect (if any).

## Author contributions

GS wrote the first draft of the paper. All authors listed have made substantial, direct and intellectual contribution to the work, and approved it for publication.

### Conflict of interest statement

The authors declare that the research was conducted in the absence of any commercial or financial relationships that could be construed as a potential conflict of interest.

## Footnotes

^1^https://www.gov.uk/government/collections/statistics-key-stage-2)
